# Evaluation of a transdiagnostic mental health intervention in German primary care: study protocol for a parallel-group, two-arm, cluster randomised controlled pilot study

**DOI:** 10.1186/s40814-025-01597-6

**Published:** 2025-01-31

**Authors:** Christopher Ebert, Marie Vogel, Jochen Gensichen, Hanna Reif, Lena Grögor, Lukas Junker, Thomas Ehring, Alkomiet Hasan, Stefan Leucht, Kirsten Lochbühler, for the POKAL-Group, Christopher Ebert, Marie Vogel, Jochen Gensichen, Kirsten Lochbühler, Markus Bühner, Tobias Dreischulte, Peter Falkai, Peter Henningsen, Caroline Jung-Sievers, Helmut Krcmar, Karoline Lukaschek, Gabriele Pitschel-Walz, Barbara Prommegger, Andrea Schmitt, Antonius Schneider, Katharina Biersack, Vita Brisnik, Julia Eder, Feyza Gökce, Carolin Haas, Lisa Pfeiffer, Lukas Kaupe, Jonas Raub, Philipp Reindl-Spanner, Hannah Schillok, Petra Schönweger, Clara Teusen, Victoria von Schrottenberg, Jochen Vukas, Puya Younesi

**Affiliations:** 1 Institute of General Practice and Family Medicine, University Hospital, LMU Munich, Munich, Germany; 2 DZPG (German Center for Mental Health), Partner Site Munich/Augsburg, Germany; 3 Department Psychology, LMU Munich, Munich, Germany; 4 Department of Psychiatry, Psychosomatics and Psychotherapy, University of Augsburg, Augsburg, Germany; 5 Department of Psychiatry and Psychotherapy, TUM School of Medicine and Health, Technical University of Munich, Munich, Germany

**Keywords:** Primary Care, Family Medicine, General Practitioner, Transdiagnostic approach, Unified Protocol, Brief Psychological Intervention, Psychoeducation, Mental Health, Pilot Study, Randomised Controlled Trial

## Abstract

**Background:**

General practitioners play an important role in the first-line care of individuals with mental health conditions. However, factors such as time constraints, limited experience in managing mental health conditions and high rates of comorbidity may hinder adequate treatment. To improve psychological care, adopting a transdiagnostic approach shows potential. Research on transdiagnostic interventions delivered by general practitioners is scarce. Thus, a transdiagnostic intervention adapted from the Unified Protocol for Transdiagnostic Treatment of Emotional Disorders was developed specifically for primary care. In a parallel-group, two-arm, cluster randomised controlled pilot study, the transdiagnostic intervention will be evaluated for feasibility, acceptability and potential effectiveness in German primary care.

**Methods:**

A total of 100 adult patients with a mental health condition will be recruited by general practitioners. In the intervention group, general practitioners will administer the transdiagnostic intervention, introducing patients to psychological concepts based on transdiagnostic factors (i.e., understanding emotions, cognitive flexibility, countering emotion-based avoidance). In the control group, general practitioners will provide improved treatment as usual oriented on official German treatment guidelines for depression, anxiety and somatoform disorders. In both study groups, treatment will be carried out in four 20-min sessions over 12-weeks. Self-report questionnaires will be completed before treatment initiation (only patients) and after treatment completion (patients and general practitioners) to assess feasibility and acceptability (i.e., treatment recruitment, delivery, response, effectiveness, unintended consequences and maintenance) as well as potential effectiveness (i.e., change in transdiagnostic factors).

**Discussion:**

The pilot study will address the research gap concerning general practitioner-led psychological interventions in primary care and will give insights into whether the adoption of a transdiagnostic approach is of benefit to general practitioners and patients. Findings may inform the design of a main trial by identifying barriers to the transdiagnostic intervention’s feasibility and acceptability, whilst advancing treatment delivery protocols to support effectiveness.

**Trial registration:**

The protocol for this study has been registered with the German Clinical Trials Register: DRKS00033386, Date of registration: 18^th^ of March 2024, https://drks.de/search/en/trial/DRKS00033386.

**Supplementary Information:**

The online version contains supplementary material available at 10.1186/s40814-025-01597-6.

## Background

Mental health conditions are highly prevalent worldwide [1]. The general practitioner (GP) is typically the first and sometimes only point of contact for affected individuals [2, 3]. However, the management of mental health conditions in primary care (PC) is often inadequate [3, 4]. This may be related to short appointment times [5] and limited training of GPs in psychological care [6, 7]. Another contributing factor could be how mental health conditions present in PC, being characterised by a less specific but rather dimensional manifestation of symptoms (e.g., various degrees of depressive and anxious symptoms) [8] and a high comorbidity rate [9–11]. Diagnosing according to ICD [12] or DSM [13] criteria and subsequently delivering a specialised treatment, as is common in the psychological and psychiatric setting, might therefore be unfeasible for GPs [3, 6, 7, 14]. To meet the contextual demands of PC [3] and to improve first-line psychological care - bridging the timespan to more specialised psychological or psychiatric services - the use of a transdiagnostic approach may be promising [6, 15].

In contrast to disorder-specific diagnosis and treatment, the transdiagnostic approach operates across or beyond diagnostic categories. The focus is thus shifted from differences between mental disorders to their similarities, so that shared mechanisms (i.e., transdiagnostic factors) can be identified and used for assessment and treatment [16]. To date, research on transdiagnostic treatments administered by GPs is scarce. The majority of previous psychological interventions in PC have taken a disorder-specific perspective [17–19]. In studies focusing on multiple mental disorders, the involvement of GPs was mostly limited to screening, while treatment was provided by others (e.g., study nurses, psychotherapists, study nurses). Only four studies were identified that focused on treating multiple mental disorders of individual PC patients and were delivered exclusively by GPs [20–23]. In two studies, GPs were trained to provide better care for stress-related mental disorders [20, 23], while GPs of the other two studies administered structured problem-solving and/or cognitive behavioural therapy (CBT) [21, 22]. The small number of studies, combined with heterogeneity in treatment methods and effects, warrants further evaluation of a transdiagnostic treatment approach in PC.

Among transdiagnostic approaches, the Unified Protocol for Transdiagnostic Treatment of Emotional Disorders (UP) [24] has the largest evidence base [25] and was successfully implemented in a wide range of settings [26]. It identifies neuroticism (i.e., the frequent experience of negative emotions) accompanied with the negative appraisal of undesirable emotions and dysfunctional forms of coping as the underlying mechanisms of “emotion-based disorders” (e.g., mood and anxiety disorders) [27, 28]. Guided by principles of CBT, the UP aims to educate individuals about emotional experiences (module 2) and introduces psychotherapeutic concepts, including mindfulness (module 3), cognitive flexibility (module 4), countering emotion-based avoidance/situational exposure (module 5/7) and interoceptive exposure (module 6) [29, 30].

To comply with recommendations for psychological interventions in PC (i.e., four to six sessions of no more than 30 min each [3]) a shortened UP-based treatment approach was developed, comprising four 20-min sessions. Previous research has shown that the delivery of a subset of UP modules, or even a single module, can already achieve symptomatic improvements [29, 31–33]. Due to the interrelated nature of the UP modules, assigning a clear therapeutic superiority to any one module is difficult [34]. Research suggests the relevance of each UP module and its targeted transdiagnostic factor across a range of mental disorders [35–37], with the exception of interoceptive exposure (i.e., confronting bodily sensations such as a racing heart), which is particularly supported for anxiety-related disorders [38, 39]. For PC, the effectiveness of cognitive reappraisal (i.e., developing greater flexibility in thought patterns) and behavioural activation/exposure (i.e., engaging in pleasurable activities/countering avoidant tendencies) as psychotherapeutic approaches has been indicated [6, 40]. Thus, considering both effectiveness and practicability, the UP modules on cognitive flexibility (module 4) and countering emotion-based avoidance (module 5) were selected as core sessions of the treatment. In addition, the UP module on understanding emotional experiences (module 2) was chosen as an introductory session to provide patients with a coherent treatment structure [41].

The objective of the cluster randomised controlled pilot study will be to evaluate the UP-based transdiagnostic intervention in German PC as compared to improved treatment as usual (iTAU). The primary aim will be to assess feasibility and acceptability in terms of patients’ and GPs’ perceptions of treatment recruitment, delivery, response, effectiveness, unintended consequences and maintenance. The secondary aim will be to evaluate potential effectiveness by monitoring patients’ trajectories in transdiagnostic factors (i.e., beliefs about emotions, experiential avoidance, emotion suppression, cognitive reappraisal and negative affectivity) as surrogate outcomes for mental health improvement.

## Methods

### Design

**Fig. 1:**
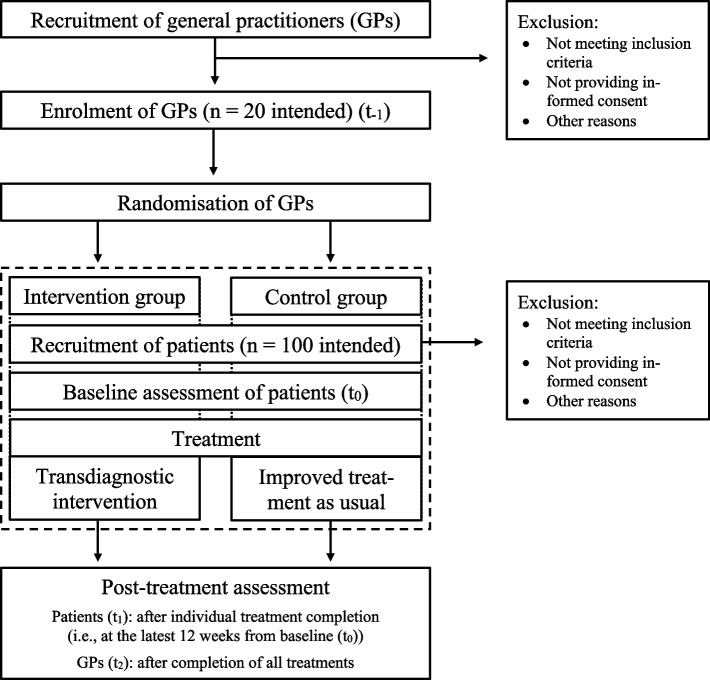
Study design and participant flow according to CONSORT

The pilot study will adopt a parallel-group, two-arm, cluster randomised controlled design. It adheres to the CONSORT statement extension to randomised pilot and feasibility trials [42] and to the SPIRIT guidelines [43] (Additional file 1). Using a 1:1 allocation ratio on PC practice level, GPs will provide patients with either a transdiagnostic intervention (i.e., intervention group (IG)) adapted from the UP [24], or with iTAU (i.e., control group (CG)) based on official German guidelines for the treatment of a depression, anxiety or somatoform disorder [44–46]. In both study groups, treatment will consist of four 20-min sessions administered over 12 weeks. Data collection will take place at baseline (patients only) and after treatment completion (patients and GPs) (Fig. 1).

### Population and setting

A total of 100 German adult PC patients with a mental health condition will be recruited by GPs from 20 PC practices within the region of Munich, Germany.

#### Eligibility criteria

Patients will be eligible if they (1) are fluent in German, (2) are at least 18 years old, (3) have a mental health condition as assessed by GPs (assisted by the Kessler-6 (K-6) [47]) and (4) provide written or digital informed consent prior to participation.

GPs will assess the following exclusion criteria by interviewing patients and reviewing their medical record: (1) Life expectancy ≤ 12 months, (2) known substance use disorder (e.g., alcohol, illegal drugs or medication; excluding nicotine dependence), (3) increased risk of suicidality, (4) having a cognitive impairment (e.g., dementia), (5) suffering from a mental disorder of great severity that warrants more intensive, specific treatment (e.g., severe depression, bipolar disorder, borderline disorder, schizophrenia, anorexia), (6) receiving psychotherapy at study start, (7) in case of psychotropic drug use, a change in medication intake in the 6 weeks prior to study start and (8) not being able to visit the PC practice for the treatment in person due to physical impairments.

GPs will be eligible if they (1) have a qualification in psychosomatic care, or alternatively have completed additional psychiatric training, and (2) provide written informed consent.

#### Recruitment

PC practices will be recruited through the teaching practice network of the Institute of General Practice at LMU Munich and with flyers sent to GPs registered with the “Kassenärztliche Vereinigung” and/or the “Bayerische Landesärztekammer”. After a GP declares their interest to participate, inclusion criteria for GPs will be confirmed in a telephone call. If a GP meets the inclusion criteria, an appointment will be scheduled in which a member of study staff will visit the practice to explain all treatment details and the procedure of the study as well as to obtain informed consent (t_-1_).

GPs will recruit study participants from their pool of new and on-going patients. The identification of patients with a mental health condition will be based on a GP’s overall impression of a patient, assisted by a short questionnaire on general psychological distress (i.e., K-6 [47]). For the latter, GPs are provided with age-specific German norm values for the K-6 [48]. Eligibility will be confirmed by GPs with a checklist, covering all inclusion and exclusion criteria.

#### Randomisation

Once at least one GP of a PC practice agrees to participate, cluster randomisation will be conducted at practice level as per a computer-generated sequence in a 1:1 ratio. This approach was chosen to prevent spillover effects within clusters, such as when several GPs from one practice participate in the study [49]. To ensure an even distribution of patients between IG and CG, randomisation will be stratified by the number of GPs in a practice providing treatment, assuming that practices with more participating GPs will treat more patients. As part of the consecutive recruitment, for each practice stratum (e.g., two participating GPs per practice), the first practice recruited is randomly allocated to either the IG or CG using the RANDBETWEEN function in Microsoft Excel [50]. This function generates an integer within a specified range (e.g., 1 or 2), with each value having an equal probability of being selected, thereby ensuring fairness in allocation. Within one stratum, the next recruited practice will be assigned to the opposite study group, and so forth. If a study group is underrepresented when a stratum is newly formed (i.e., more practices have been assigned to either study group overall), this study group will be prioritised for allocation to prevent imbalance in the distribution of practices.

Randomisation will be managed and documented by a staff member of the Institute of General Practice at LMU Munich, who is not part of the study [50]. Allocation concealment will be maintained using opaque envelopes, which will be opened by study staff only shortly before GPs are trained in their assigned treatment condition.

#### Blinding

Due to the design of the study, it is not possible for GPs, patients or study staff to be blinded to the study group allocation. However, participants will be blinded to which of the two treatments is considered the active intervention.

#### Sample size

No formal sample size calculation is required for a pilot study [51]. To minimise the maximum likely error for any calculated rates to less than 10%, Eldridge et al. [52] suggested a total of 23 clusters for cluster-randomised pilot studies with a cluster size of five participants, assuming an intra-cluster correlation (ICC) of 0.05. Similar ICCs were found for psychological outcomes in German PC [53] and recruiting six patients per PC practice was considered feasible for psychological interventions [54, 55]. To balance scientific considerations and practical constraints (i.e., time and staff resources), a sample size of 100 patients across 20 PC practices is anticipated, with six patients enrolled per practice, accounting for an expected 20% dropout rate. This should provide sufficient data to adequately assess treatment feasibility, acceptability and potential effectiveness, forming a robust basis for a confirmatory main trial.

### Procedure

Before treatment start, IG and CG practices will receive a two-hour training (t_-1_) in which GPs are informed about the structure and content of the treatment (i.e., transdiagnostic intervention or iTAU). In both study groups, upon obtaining informed consent and before the first treatment session is delivered, patients will complete a baseline questionnaire (t_0_) in their PC practice via tablets using the online platform SoSci Survey [56]. In case of technical problems, a printed version of the baseline questionnaire will be used. No later than 10 days after completion of the baseline questionnaire, participants will receive the first session of their allocated treatment. Over the course of the 12-week treatment, selected study parameters (e.g., treatment fidelity, serious adverse events) of patients will be recorded with a documentation sheet by GPs. After the last (i.e., fourth) treatment session, a post-treatment digital questionnaire (t_1_) will be provided to patients which will again be completed in their PC practice with tablets (alternatively, a printed version will be used). Once the treatment of all patients is completed, GPs will be invited to fill in a digital questionnaire (t_2_). Study participation is free of charge. As compensation and to promote participant retention, participants will receive a 25€ voucher after completion of each questionnaire. GPs will be incentivised with 100€ for each recruited patient.

#### Treatment

Intervention condition: An adapted version of the UP [24] will be administered by GPs to patients. The treatment consists of four 20-min sessions. To facilitate treatment delivery, a table-flipchart was designed, comprising session-specific slides. In session 1, GPs introduce the functional model of emotional processing which identifies negative reactivity to emotions and dysfunctional coping mechanisms as common factors for emotion-based disorders. In addition, the function and composition of emotions are explained. Session 2 addresses the concept of cognitive flexibility. The interrelationship of thoughts and feelings is especially emphasised. Patients will be encouraged to question automatic negative thoughts and to adopt flexibility in their cognitive appraisal. Session 3 focuses on emotion-based avoidance. Different types of emotion-based avoidance are discussed and possible alternative actions are explored to enable an active and constructive processing of undesirable emotions. In session 4, GPs and patients reflect on the course of treatment, summarise major takeaways and discuss further (psychotherapeutic) treatment options, if necessary. To complement the treatment, patients will be given a booklet. After each session, patients will receive a prescription from their GP to complete a corresponding section in the booklet. This involves reading a text (e.g., concept of cognitive flexibility), completing a related task (e.g., reflecting on automatic thoughts during an emotionally stressful situation) and engaging in two enjoyable activities (e.g., taking a walk in nature, talking to a friend).

Control condition: In the CG, iTAU will be delivered. GPs are instructed to align treatment with the official German guidelines for the treatment of a depression, anxiety or somatoform disorder [44–46]. Apart from carrying out four 20-min sessions, no further structure is provided.

#### Risk management

No negative side effects are to be expected for study participants. Patients with a severe mental health condition (e.g., suicidal ideations) will be identified by GPs at study start and provided with the necessary, more specific care. In the event of a serious adverse event, GPs will ensure their patient’s well-being, decide whether continuation in the study is possible and communicate this to the study team.

### Outcomes

Feasibility, acceptability (i.e., primary outcomes) and potential effectiveness (i.e., secondary outcome) will be assessed quantitatively. A digital questionnaire will be answered by patients at baseline (t_0_) and at the end of their treatment (t_1_). GPs will be surveyed after completion of all treatments (t_2_) (Table 1). Due to the uncertainty of estimates in pilot studies, no definitive thresholds were defined for proceeding to a confirmatory main trial [42]. Rather, the decision to proceed will be based on the overall implications of the intervention’s feasibility, acceptability and potential effectiveness, the criteria for which are outlined below.

**Table 1:**
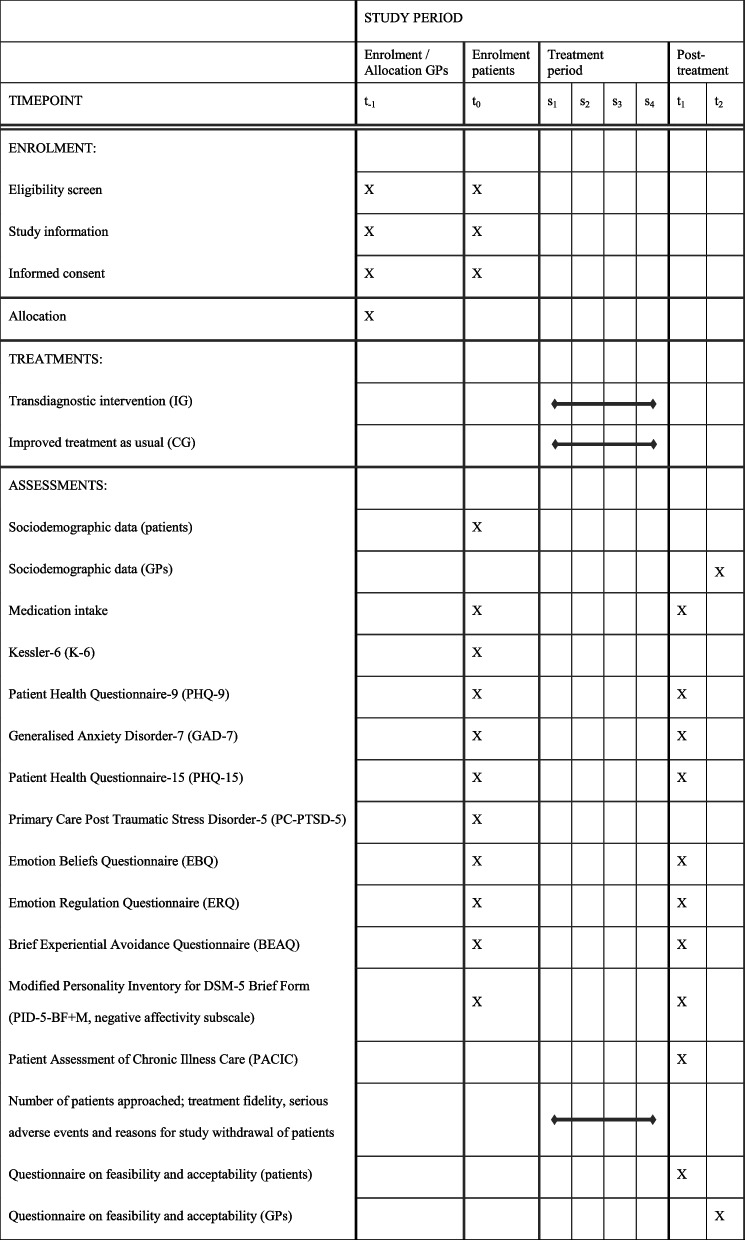
Schedule of enrolment, treatment and assessments according to SPIRIT

s1, s2, s3, s4 = treatments sessions 1 - 4; GPs = general practitioners; IG = intervention group; CG = control group

#### Primary outcome measures

Feasibility and acceptability of the intervention and control treatment will be evaluated post-treatment (patients: t_1_; GPs: t_2_). Corresponding questionnaires are based on the process evaluation framework for cluster randomised controlled trials of Grant et al. (2013) [57] and a previous similar pilot study conducted in the PC setting [58]. Patients and GPs will be asked to respond to statements on treatment recruitment, delivery, response, effectiveness, unintended consequences and maintenance. Their degree of agreement will be assessed on a 4-point Likert scale (“strongly disagree” to “strongly agree”). Examples of questions for GPs and patients are: recruitment- “Recruited patients did not differ from patients who did not participate”; delivery - “I followed the instructions for implementing the treatment”; response - “The content of the treatment sessions was understandable to me”; effectiveness - “The treatment helps patients to better manage their mental health condition”; unintended consequences - “The treatment did not lead to any undesired consequences for me”; maintenance - “I would recommend the treatment to my colleagues“. For additional questions on the duration and amount/number of treatment sessions, 3-point Likert scales will be used (“too short”, “adequate duration”, “too long”; “too few”, “adequate amount/number”, “too many”, respectively). The overall treatment satisfaction will be monitored in school grades (1: “very good” to 6: “very bad”).

#### Secondary outcome measures

At t_0_ and at t_1_, patients will complete self-report diagnosis-specific and transdiagnostic questionnaires. Potential effectiveness will be based on change in transdiagnostic factors relevant to emotion-based disorders [27, 28], thus serving as surrogate outcomes [42] for patients’ improvement in mental health.

##### Diagnosis-specific outcomes

Patient Health Questionnaire-9 (PHQ-9): The PHQ-9 [59] assesses the occurrence of depressive symptoms over the last two weeks with nine items rated on a 4-point Likert scale (0: “not at all present” to 3: “present nearly every day”). The total score can range from 0 (no depression) to 27, with larger scores being indicative of greater dysfunction. Cut-offs of ≥ 5, ≥ 10, ≥ 15 and ≥ 20 can be applied to differentiate between a mild, moderate, moderately severe and severe form of depression. The German PHQ-9 was shown to have satisfactory internal consistency (α = 0.89) in a PC sample as well as to fulfil construct and criterion validity [59].

Generalised Anxiety Disorder Screener‐7 (GAD-7): The GAD-7 [60] is a 7-item scale to measure general anxiety symptoms within the past two weeks on a 4-point Likert scale (0: “not at all present” to 3: “present nearly every day”). Total scores can range from 0 (no anxiety) to 21. A larger score represents a more severe impairment. Mild, moderate and severe anxiety symptoms are associated with a score of ≥ 5, ≥ 10 and ≥ 15, respectively. In a representative sample of the German population, the internal consistency of the GAD-7 was found to be satisfactory (α = 0.89) and its construct validity has been shown [60].

Patient Health Questionnaire-15 (PHQ-15): The PHQ-15 [61] uses 15 items rated on a 3-point Likert scale (0: “not bothered at all” to 2: “bothered a lot”) to determine somatic symptom severity experienced over the last four weeks. The sum score can vary between 0 (no somatic symptoms) and 30. Higher values correspond with an increased probability of suffering from a somatoform disorder. Cut-off points for low, medium and high somatic symptom severity were established for values ≥ 5, ≥ 10 and ≥ 15. The internal consistency (α = 0.80) as well as convergent and discriminant validity of the German PHQ-15 in a PC sample were deemed satisfactory [61].

Primary Care Post Traumatic Stress Disorder-5 (PC-PTSD-5): The PC-PTSD-5 [62] evaluates post-traumatic stress symptoms in the last 30 days with five dichotomous items (occurrence: “yes”/”no”). The total score can range from 0 (= no post-traumatic stress) to 5 and a score of 3 has been set as a psychopathological threshold. In a PC sample, the PC-PTSD, consisting of the first four items of the PC-PTSD-5, demonstrated good test–retest reliability (r = 0.83) and predictive validity [63].

##### Transdiagnostic outcomes

Emotion Beliefs Questionnaire (EBQ): The EBQ [64] measures affective mentalisation which entails the perceived controllability and usefulness of positive and negative emotions, and thus corresponds with the content of the first session of the transdiagnostic intervention. The EBQ comprises 16 items rated on a 7-point Likert scale (1: “strongly disagree” to 7: “strongly agree”). Eight items measure “general-controllability”, four items each examine “negative-usefulness” and “positive-usefulness”. The sum score can vary between 16 and 112, with higher values indicating that positive and negative emotions are perceived as less controllable and more useless. In a German non-clinical sample, the EBQ showed satisfactory internal consistency (α = 0.87), as did the subscales (general controllability: α = 0.85; negative-usefulness: α = 0.76; positive-usefulness: α = 0.70). Convergent and discriminant validity were also demonstrated [65].

Emotion Regulation Questionnaire (ERQ): The ERQ [66] assesses emotion regulation with ten items, of which four items measure emotion suppression and six items cover cognitive reappraisal. Emotion regulation is the primary skill targeted in the transdiagnostic intervention, with the reappraisal subscale closely aligning with the content of the second session. All items of the ERQ are rated on a 7-point Likert scale (1: “strongly disagree” to 7: “strongly agree”). Total scores on the suppression and reappraisal subscales can range from 4 to 28 and 6 to 42, respectively. Higher values on each subscale indicate a greater preference for suppression or reappraisal. The German ERQ reached satisfactory internal consistency for both the suppression (α = 0.74) and reappraisal (α = 0.76) subscale in a sample of medical students. Further, convergent and discriminant validity were indicated [67].

Brief Experiential Avoidance Questionnaire (BEAQ): The BEAQ [68] comprises 15 items rated on a 6-point Likert scale (1: “strongly disagree” to 6: “strongly agree”). It matches the content of the third session of the transdiagnostic intervention as it measures a tendency towards emotion-based avoidance. A higher overall score, varying between 15 and 90, suggests a greater extent of experiential avoidance. With the German BEAQ, five dimensions of experiential avoidance can be monitored: behavioural avoidance, distress aversion, procrastination, distraction/suppression and repression/denial. In a mixed sample of students and outpatients, the BEAQ yielded satisfactory internal consistency (α = 0.80) and test–retest reliability (r = 0.77). Discriminant and convergent validity were also shown [69].

Modified Personality Inventory for DSM-5 Brief Form (PID-5-BF + M): The PID-5-BF + M [70] assesses the personality traits of psychoticism, disinhibition, detachment, antagonism, anankastia and negative affectivity. Only the latter subscale will be employed in the pilot study, as negative affectivity (i.e., neuroticism) is the core transdiagnostic concept upon which the UP was developed [71]. Negative affectivity is measured with six items on a 4-point Likert scale (0: “very false or often false” to 3: “very true or often true”). Ranging between 0 and 18, a higher subscale score suggests greater negative affectivity. Norm values for the PID-5-BF + M were established for the German population [72]. In a PC sample, satisfactory reliability scores (α = 0.68—0.81) as well as convergent and discriminant validity were observed [73].

Kessler Psychological Distress Scale-6 (K-6): The K-6 [47] comprises 6 items that examine psychological distress over the last 30 days. Responses are given on a 5-point Likert scale (0: “none of the time” to 4: “all of the time”). The total score can range from 0 (no psychological distress) to 24, with higher values suggesting more distress. Commonly used in the PC setting [74], the K-6 will only be administered at t_0_ to complement GPs’ judgment of a patient’s psychological distress level. Established age-specific norm values for the German population will be provided to GPs [48]. The German Kessler-10, a longer version of the K-6 with four additional items, was found to have high internal consistency (students: α = 0.80; outpatients: α = 0.90) as well as satisfactory convergent validity [75].

#### Additional outcomes

At baseline (t_0_), socio-demographic data (i.e., age, sex, nationality, material status, highest educational level, current employment status, previous psychiatric diagnosis and corresponding treatment) will be assessed from patients. Patients’ medication intake will be recorded at t_0_ and t_1_. Also, at t_1_, the quality of treatment received for chronic illnesses of patients over the last 6 months will be examined, if applicable. For this, the Patient Assessment of Chronic Illness Care (PACIC) short form [76] will be used, which consists of 11 items rated on a 4-point percentage scale (0–25% to 76–100%). Over the course of the treatment period, GPs will document the number of patients approached for the study as well as treatment fidelity, any serious adverse events and reasons for study withdrawal of recruited patients. Among GPs, socio-demographic (i.e., age, sex) and occupation-related data (i.e., work experience, additional training, certain characteristics of their practice) will be collected post treatment (t_2_).

### Statistical analysis

Quantitative data analysis will be conducted using R [77]. Descriptive statistics will be reported for the samples of patients and GPs (e.g., socio-demographics, treatment fidelity, characteristics of recruited PC practices). Scores for feasibility and acceptability items will be presented graphically for the IG and CG treatment groups, along with patients’ trajectories in both transdiagnostic and disorder-specific outcomes. The potential effectiveness of both treatment conditions will additionally be estimated via linear mixed models with restricted maximum likelihood estimation (REML), known to perform well with small sample sizes [78]. Changes over time in patients’ beliefs about emotions (EBQ), emotion suppression /cognitive reappraisal (ERQ), experiential avoidance (EBAQ) and negative affectivity (PID-5-BF + M) will be examined. Treatment group (IG, CG), time (t0, t1) and their interaction will be included as fixed effects. To account for the assumed ICC of recruited PC practices, patients’ practice affiliation will be modelled as a random effect, with the interaction between practice and time specified as a random slope. Changes over time in patients’ symptoms of depression (PHQ-9), anxiety (GAD-7) and somatoform disorder (PHQ-15) will also be analysed using linear mixed models.

To avoid missing values for digitally collected data, all questions in SoSci Survey [56] must be answered before completing data entry. Missing values for partially completed (printed) questionnaires will be imputed if deemed necessary. As the pilot study will not be adequately powered to assess treatment effectiveness, for all statistical tests, only the confidence interval limits will be reported without relying on the *p*-value [42, 79].

### Data collection and management

Patient recruitment started on June 1^st^ 2024 and is scheduled until March 31^st^ 2025, with data collection to be completed by June 30^th^ 2025. Participants will be enrolled by GPs consecutively, at the latest 12 weeks prior to the end of the data collection period. Data of patients (t_0_ and t_1_) and GPs (t_2_) will be collected using the online platform SoSci Survey [56] and stored on a secure internal server of the Institute of General Practice at LMU Munich. Data collected by printed questionnaires will be securely stored at the study site, with access restricted to study staff. The sociodemographic, health and treatment assessment related data will be collected in pseudonymised form. Patients create their own pseudonym based on predefined criteria (e.g., the first letter of their mother’s and father’s name) which has to be entered t_0_ at and t_1_. GPs are assigned one numerical code per practice to be used at t_2_. Personal data (e.g., name, telephone number) which is requested from GPs and patients as part of obtaining informed consent will be stored separately from their sociodemographic, health and treatment related data. Two lists are kept at the Institute of General Practice at LMU Munich documenting the names of patients/GPs and their pseudonyms. Both lists are protected by technical and organisational means against unauthorised third-party access. Decoding will only take place in the event that contacting patients for data collection outside of their PC practice becomes necessary, patients’ safety requires it (i.e., medical reasons) or the scientific question changes (i.e., scientific reasons). For decoding data due to scientific reasons, the approval of the ethics committee at LMU Munich is obtained beforehand.

No data monitoring committee will be installed due to the unblinded, non-invasive, short-term character of the pilot study. Further, GPs and patients can request the deletion of their stored data at any time. Person-identifying data will be deleted after the end of the research project, or as soon as the study objective has been reached. The remaining anonymised data will be stored for at least 10 years and deleted at the latest 15 years after their acquisition unless legal requirements stipulate longer archiving obligations.

## Discussion

This study protocol outlines the design of a parallel-group, two-arm, cluster randomised controlled pilot study to evaluate a UP-based transdiagnostic intervention compared to iTAU in German PC. Feasibility and acceptability will be assessed as primary outcomes and potential effectiveness as a secondary outcome. The rationale for a transdiagnostic intervention in PC is guided by the existing challenges in managing mental health conditions in this setting [3, 6, 8]. Although providing psychological support is considered an important component of PC, its realisation often appears to be challenging or even inadequate [4, 7]. Contributing factors include time constraints [5], GPs’ lack of experience in managing mental health conditions [9–11] and high rates of comorbidity [11]. To date, research on psychological interventions administered by GPs, especially those using a transdiagnostic approach, is limited [6, 17–19]. The pilot study aims to address this research gap.

The adaptation of the transdiagnostic intervention from the UP ensures a sound evidence base. As the most researched transdiagnostic approach [25], the UP has been shown to be applicable to a variety of settings and patient groups [26]. In order to be administered in the PC setting, the UP was shortened. While this may affect its effectiveness [80], previous research demonstrated that a condensed version of the UP still leads to symptomatic improvements in patients [29, 31–33]. Furthermore, the selection of UP modules for the pilot study was based on psychotherapeutic concepts identified as effective for common mental disorders in PC [6, 40]. Concerning the CG, choosing iTAU as a treatment condition permits some degree of standardisation while not compromising external validity [7].

For practicability, the pilot study will be limited to PC practices within the region of Munich, Germany. In addition, although the sample size does not allow definitive conclusions to be drawn about the effectiveness of the intervention [51], a target of 100 patients is reasonable for a pilot study [81]. This should allow for an adequate analysis of feasibility, acceptability and a preliminary assessment of effectiveness, providing a solid rationale for a larger main trial [52, 82]. It is also important to note that, in line with the stepped care model [83], the goal of the transdiagnostic intervention is not to resolve a patient’s mental health condition, but rather to provide initial support and bridge the gap until specialised psychological or psychiatric services are available, if needed. In general, psychological counselling in PC has been shown to produce small short-term effects [7].

Should the transdiagnostic intervention prove feasible, acceptable and potentially effective, several implications arise: First, the transdiagnostic intervention should be refined based on the pilot study findings and tested in a larger main trial. Second, research on the application of a transdiagnostic approach in the PC setting should be expanded. This could include exploring its integration with precision psychiatry by tailoring the delivery of transdiagnostic modules to patients’ individual needs [32, 33]. Beyond these steps, future research could investigate the long-term outcomes of transdiagnostic interventions in PC and assess their cost-effectiveness compared to disorder-specific treatments. Ultimately, the pilot study will contribute to improving the treatment of PC patients with mental health conditions.

## Supplementary Information

Additional file 1. SPIRIT 2013 Checklist.

## Data Availability

The anonymised dataset and statistical code supporting the conclusions of the pilot study will be made available in the Open Science Framework (OSF) repository (https://doi.org/10.17605/OSF.IO/96U34) when the pilot study is completed and published.

## Abbreviations

BEAQ: Brief Experiential Avoidance Questionnaire
CBT: Cognitive Behavioural Therapy
CG: Control Group
DSM: Diagnostic and Statistical Manual of Mental Disorders
EBQ: Emotion Beliefs Questionnaire
ERQ: Emotion Regulation Questionnaire
GAD-7: Generalised Anxiety Disorder Screener-7
GP: General Practitioner
ICC: Intra-Cluster Correlation
ICD: International Classification of Diseases
IG: Intervention Group
iTAU: Improved Treatment as Usual
K-6: Kessler Psychological Distress Scale-6
PACIC: Patient Assessment of Chronic Illness Care
PC: Primary Care
PC-PTSD-5: Primary Care Post Traumatic Stress Disorder-5
PHQ-15: Patient Health Questionnaire-15
PHQ-9: Patient Health Questionnaire-9
PID-5-BF + M: Modified Personality Inventory for DSM-5 Brief Form
UP: Unified Protocol
